# The Study of the Diseases of the Eye

**Published:** 1838-01

**Authors:** 


### Art. II.

1. *Lehre von den Augenkrankheiten. Zum Gebrauche für practische Aerzte und Wundärzte, so wie zur Benutzung als Leitfaden beim klinischen Unterrichte, abgefasst.* Von Dr. Anton Rosas.—*Wien*, 1834. 8vo. pp. 599.

*The Study of the Diseases of the Eye, for the Use of Medical Practitioners and for Clinical Instruction.* By Dr. Anton Rosas.—*Vienna*, 1834. 8vo. pp. 599.

2. *Dell' Ottalmoscopia e dell' Introduzione allo Studio dell' Ottalmologia. Dissertazione del* Dottore Luigi Marchetti da Cremia.—*Pavia*, 1834.

*A Dissertation on Ophthalmoscopy, and an Introduction to the Study of Ophthalmology.* By Dr. Marchetti.—*Pavia*, 1834. 8vo. pp. 207.

3. *De l'Emploie de l'Excision et de la Cauterisation dans l'Ophthalmie blennorrhagique.* Par E. F. Julliard.—*Paris*, 1835. 4to. pp. 88.

*On the Use of Excision and Cauterization in purulent Ophthalmia.* By E. F. Julliard.—*Paris*, 1835. 4to. pp. 88.

4. *The Cyclopædia of Practical Surgery.* Edited by W. B. Costello, m.d. Part I. Art. *Amaurosis*, by Fred. Tyrrell, Esq.—*London*, 1837. 8vo. pp. 112.

5. *A Manual of the Diseases of the Eye.* By S. Littell, Jun. m.d.—*Philadelphia*, 1837.—8vo. pp. 255.

There is no fact more striking in the annals of medicine than the rapid strides towards perfection which have been made in the domain of Ophthalmology within less than the last half century; and it may be justly affirmed that the most important epoch in its history dates from the foundation of the Imperial School of Vienna. Austria has the merit not only of having been the first to establish an academy for the advancement of ophthalmic science, but of having based it on liberal and rational principles; and, among a people so distinguished for zeal and industry, no sooner was an impulse given, than men, alike distinguished as sagacious observers and faithful interpreters of nature, succeeded not only in raising from ignominious abasement this department of science, but in imparting to it a degree of precision, and advancing it to a pitch of perfection, which no other branch of the healing art can even yet be said to have reached. Schmidt and Beer stand forth preeminent among those who first attained an exact knowledge of ophthalmic diseases. Setting aside all preconceived notions and theories, they never stepped beyond the boundaries of strict observation, nor permitted themselves to enter into the visionary and delusive speculations that then pervaded German medicine. They chose neither to believe nor to assert where evidence was wanting. They knew that to paint things as they really are requires minute attention: hence, their descriptions of disease, copied from nature, have all much of the freshness and liveliness produced by actual perception. The exquisite tact they evinced in discriminating the shades of organic lesion was acknowledged by their contemporaries, and their prelections and clinics were frequented by a concourse of students from every civilized country. A death-blow was thus given to the spirit of empiricism, which then prevailed to an immoderate extent. For it may be truly said, that, until the beginning of the present century, the prerogative of treating the diseases of the eye had been in a great measure usurped by quacks and itinerant impostors.

The impulse being thus given, ophthalmic institutions sprang up in other countries. Skilful practitioners undertook the discharge of the important duties attached to them; and, as they pursued the same line of research, they proved greatly instrumental in extending still further the useful applications of this branch of medical science.

If slow to enter on this career, and although certainly still behind Germany in the general knowledge of ophthalmological science, England must now be admitted to have been for some time actively and effectively engaged in cultivating this most interesting and important branch of surgery. The last few years have seen produced in this country more works exclusively dedicated to the pathology of the organ of vision than were published during the whole of the last century. The reason of this is obvious; this class of maladies is no longer the exclusive property of the so-called oculist, but, on the contrary, forms an integral part of the knowledge of the well educated physician or surgeon. Five and twenty years ago the profession, especially in the provinces, was avowedly and lamentably ignorant of the first principles of this branch of medical science; while at the present day, from the increased facilities afforded by the numerous ophthalmic hospitals in the metropolis and in most of the large country towns, the study of diseases of the eye forms no unimportant branch of the labours of the student, although, perhaps somewhat unwisely, it forms as yet no part of the examinations at the chartered medical colleges.

In the present Article we purpose to pass in review a few of the works on diseases of the eye which have been gradually accumulating on our hands, noticing, at any length, such matters only as appear particularly interesting or are of much practical utility.

Comprising, as it does, all the organic textures distributed throughout the body, besides some peculiar to itself, as the cornea, iris, choroid, and retina, the eye has been truly represented as a sort of miniature or recapitulation of the whole animal fabric. Thus, for example, the osseous system is present in the bony shell of the orbit; the dermoid system, in the palpebral integument; the mucous, in the conjunctiva and lachrymal apparatus; the serous, in the cornea, in the choroid, iris, lens, and hyaloid membrane; the fibrous, in the sclerotic, the sheath of the optic nerves, and periorbital investment; the vascular, in the arteries, veins, and more especially in the choroid; the muscular, in the muscles of the eyelids, eyeball, lachrymal organs, and, according to some authors, in the iris; the glandular, in the conjunctiva, the meibomian follicles, the lachrymal gland and caruncles; lastly, the nervous system, in the ophthalmic nerves and retina. It is accordingly found that the eye is subordinate to the same physiological laws, and liable to the same morbid influences, as other parts of the body. Diseases of a strictly peculiar kind cannot, therefore, be said to exist in the eye: all its affections being more or less identical with those of the rest of the organized tissues; springing from the same causes, modified by the same principles, whether general or specific, and exhibiting the same phenomena. Hence, scrofula, rheumatism, gout, catarrh, syphilis, the exanthemata, dropsy, blennorrhœa, present similar phases, observe similar stadia and select similar textures for their seat in the eye, as in other parts of the body. Indeed the further we prosecute this train of investigation, the more abundantly and strikingly will analogies present themselves. Every successive step will but corroborate the soundness of that scheme which teaches us to regard the eye not as an isolated or peculiar structure; and that the treatment of its diseases, in order to be rational, must repose on the same bases as that of other distempers.

I. The first work on our list, that of Professor Rosas, is rather a compendium than a complete treatise; it comprehends the pathology and treatment of most of the diseases of the eye. The arrangement of its contents is on the whole good and in some respects original, although contaminated with the besetting sins of German systematic writers, the love of infinitesimal division, the splitting of species into innumerable subspecies, and the introduction of the most elaborately-compounded words of Greek extraction to express the simplest phenomena.

The author's descriptions of diseases are generally accurate, and his rules for surgical manipulation clear and simple; but his method of treatment is often faulty, from the mass of obsolete drugs with which it is complicated.

The author commences his work by detailing the different affinities subsisting between the eye and the other organs and systems of the body. Among these the principal are, 1st, its connexion with the remaining organs of sense; 2d, with the brain; 3d, with the viscera of the abdomen; 4th, with the skin; 5th, with the generative system. Most of these sympathies he attempts to explain by the physical connexions of the nerves of the different parts, but unsuccessfully of course. We know that sympathies, for the most part, depend on a more general cause. The influence of congestion and disordered function of the abdominal viscera in determining or modifying ophthalmic affections is well known. Saburral conditions of the primæ viæ, excess or acrimony of the biliary secretion, or the presence of intestinal worms, prove the fruitful source of disease in remote organs, and, among others, of the eye. Venous congestion in the abdomen produces like effects. This is shown by the fact often witnessed that where a load of recrementitious matter has been removed by purgatives, ophthalmic diseases have promptly subsided.

The sympathies that have been traced between the organs of sight and respiration have been overlooked by the author. They are, however, often very remarkable. Jüngken tells us that he was acquainted with "two persons so habituated to the stimulus of light that its presence became indispensable towards the due performance of respiration, insomuch that they were instantaneously seized with asphyxia when they found themselves in a place from which light was excluded; and suddenly awakened from sleep with a sense of approaching suffocation if by any chance the tapers in their chamber came to be extinguished."* This case strikingly resembles one recorded by Laennec: a nobleman, in whom attacks of

* Die Lehre von den Augenkrankheiten, p. 12.

asthma invariably came on when any person inadvertently shut his bed-room door, or his night-lamp happened to go out.*

The sympathy between the visual and generative organs is often remarkable. A gonorrhœa suddenly suppressed frequently excites the most virulent ophthalmia; affections of the testis and uterus are fertile sources of diseases of the eye; and, conversely, some of the most intractable forms of such diseases yield on the establishment of the catamenia, or are quickened into activity on the cessation or interruption of that function.

In the author's history of individual diseases, many things are well worthy of notice both for their novelty and their importance. Under the head of *Neuroses* we find the following account of "Iridalgia," which, however, we must greatly abridge.

"It is a painful affection, seated in the ciliary nerves of the iris, and extending to the contiguous nerves. The individuals most liable to its attacks are those whose eyes are much engaged in reading, writing, or contemplating minute and brilliant objects; or who require to make frequent transitions from an obscure or highly illuminated place. Hence, literary men, lace-workers, printers, especially compositors, are chiefly subject to this disease. Repletion favours the supervention of a paroxysm, which is for the most part confined to one eye. The following are the symptoms. At the instant of seizure the patient finds himself dazzled, and the central point of any object that may be present to him seems enveloped in mist. He next observes a black spot, which gradually enlarges and after the lapse of a minute or two appears encircled with a transparent, coloured, or pale halo, having a zig-zag oscillatory movement. This zone, at the beginning narrow, becomes bigger and bigger in proportion as the mid-point gets distinct, and emitting sparks progressively advances to the circumference of the iris, when it vanishes. Although the eye be open or closed, the phantasm is always present, with this difference that it is more marked in twilight or in the dark. The paroxysm varies in point of duration. The spectrum lasts from a few minutes to half an hour. Up to this time and immediately after evanescence no particular pain is felt, but a sort of obtuseness, troubled sight, and weight in the head, in some instances a degree of dazzling and cloudy vision, are the sole precursors of the neuralgia, and these so trivial as hardly to attract the patient's attention.

"On the subsidence of the spectral illusion, transient darts of pain are experienced in the eye and corresponding temple; the whole eyeball next becomes uneasy, and the least pressure occasions sudden sharp pain. There is a feeling of tension, fulness, and violent compression, as if under the blow of a hammer. The greatest suffering is in the superior and internal parts of the eye, and is not uniform, but observes remissions and exacerbations, without entirely ceasing, augmenting in intensity about every ten or fifteen minutes and persisting several hours or even days. Vision, smell, hearing, taste, are all during the paroxysm dulled; the eyes are red and suffused, the pupil is contracted. Exposure to light renews or aggravates the fit. The slightest noise is intolerable. There is anorexia and sometimes vomiting; disposition to drowsiness; but the intellect remains clear."

There is nothing particular in the treatment recommended. Darkness and quietude, mild narcotics internally and locally, attention to the state of the stomach, and bloodletting if there is plethora, are the chief measures.

Dr. Rosas has furnished some useful directions in reference to the best modes of applying galvanism in paralysis of the eyelid or "Blepharoplegia." He insists particularly on the superior efficacy of the negative over the positive pole in the treatment of paralysis.

* Translation of Laennec, p. 414.

"In order to stimulate the ophthalmic nervous apparatus through the medium of the fifth pair, the negative pole is to be connected with one or other of the branches of that nerve in the proximity of the supra-orbital notch, the inner surface of the nose or upper jaw, or the temples. To secure coaptation, a somewhat concave metallic disc may be employed: it must be gilt within, to prevent oxidation, and lackered without and over its rim, that it may not touch the skin, and must have in its concavity a bit of thin sponge, moistened with some saline solution, fixed by a band drawn through the disc, so as to compress it against the part to be galvanized, and fastened by an eye to the wire of the negative pole. From ten to twenty-five pairs of plates are required.

"The physiological effects of that modification of the application of voltaic electricity, called the 'galvanic puncture,' are as follows:—So soon as the circle is complete, or the wire of the battery in communication with the needle, the patient experiences proportionately to the intensity of the action, a violent, darting, tearing pain, felt not only during the period of contact but for many hours after, though in a lower degree." . . . "A contraction and oscillation of the adjacent muscles is another effect of the galvanic puncture. The surrounding skin and the portion intermediately betwixt the points of insertion alternately corrugates into small folds. And we observe in a little while, about the points of insertion, a circumscribed red areola of from two to three lines diameter." . . . "Where the zinc pole acts, the inflamed halo rapidly appears; the integument becomes hard and of a bright scarlet; and there issues from the puncture at first watery serum, but afterwards thick and turbid lymph. On the other hand, a conical bluish-grey elevation surrounds the needle connected with the negative pole, which contains air diffused throughout minute cells, bursting with a crackling noise after the withdrawal of the needle. Here a plain surface covered with the raised but not discoloured integument remains behind, while in the former case the surface is for a considerable time, hard, prominent, and red." . . . "After the lapse of three or four days we find in the above situations circular sores of from one to two lines deep. The ulcers determined by the zinc pole have a redder margin and are more inflamed than the others."

The galvanic puncture is considered more energetic in its action than the simple shock, inasmuch as the needle in union with one or other pole, by penetrating the flesh, comes into closer contact with the nerve.

In operating for cataract of late years, the superior has been preferred to the inferior section of the cornea. This mode is attended with less risk of protrusion of the iris or discharge of the vitreous humour, and is seldom followed by much vascular reaction. But, on the other hand, if the cornea be relaxed and flaccid, it is difficult, nay sometimes impossible, to procure union of the edges of the flap. Dr. Rosas recommends the *external lateral* section in consequence of the favorable result of twenty-one operations performed partly with Beer's knife and partly with his own two-edged knife. He does not consider so limited a number of trials sufficient to warrant the general introduction of this method into practice; but leaves its merits to be decided by subsequent experience.

In cases of inveterate opacity of the cornea and which have resisted all ordinary remedies, the author proposes, in addition to the artificial enlargement of the pupil, or the insertion of a seton through the cornea by means of a finely-polished curved needle, as suggested by Pellier, the inoculation of the matter of blennorrhœal ophthalmia. In this way the obscure cornea is occasionally restored to its normal transparency. We must, however, regard inoculation as a very hazardous experiment. The difficulty that is met with in restraining the violence of so distressing a disease as purulent ophthalmia, independently of the alarming structural changes incidental to it, are obstacles too serious to counterbalance any probable good that may ensue, more especially as the formation of artificial pupil supplies an almost infallible and comparatively safe means of counteracting the defect in question.

It was formerly a generally received opinion among ophthalmological writers, that penetrating wounds of the lenticular capsule are necessarily followed by cataract. This is true, however, only in reference to lacerated wounds. Dr. Werneck not long ago instituted a series of experiments on the eyes of living animals, for the purpose of ascertaining the effect of wounds of the capsule and lens. Among other results Dr. W. found that if the outer lamella of the capsule be punctured, incised, or even cauterized, no scar is left; nor even when both lamellæ, along with the membranous coat of the lens, are pierced. But that in every instance of laceration of the fibres a permanent opaque spot was the invariable consequence. Dr. Rosas, in describing wounds of the lens, corroborates the above conclusion.

"When wounds go deeply into the lens, or when the anterior segment of the capsule is cut in several places or torn across, so much more certainly is cataract developed. Should the iris and ciliary body have been simultaneously injured, then is the accident complicated with exudation of lymph and adhesions of adjacent parts."

We conclude our notice of Dr. Rosas' book with the abstract of a remarkable case of aneurism of the orbit discovered during life, and the only one ever witnessed by the author.

"The subject, a girl eighteen years of age, had previously laboured under scrofulous disease, and irregular menstruation. The aneurismal affection was imputed to a severe blow near the eye. She complained of frequent dull pain in the bottom of the right orbit, from whence the eye to a certain extent protruded, but might still be covered in a great measure by the eyelids. No morbid change of structure could be detected. Any undue determination of blood to the head produced redness of the eye with a sense of heat and throbbing in the orbit, vertigo, tinnitus aurium, and imperfect vision. These symptoms were always present on the approach of the catamenia, which were scanty and irregular. On examination, a distinct pulsation might be felt deeply seated in the orbit along with a thrilling murmur. Repeated venesection in the foot; leeches to the genitals; stimulant foot-baths; and emmenagogue medicines, restored the faulty menstruation. By antiphlogistic regimen, cold applications to and occasional leeching of the eye, the aneurismal disease was much relieved."

II. In the work of Dr. Marchetti we find a critical investigation and arrangement of whatever has been done in modern times for general *ophthalmoscopy*. It is, as its title implies, a guide-book for the examination of the diseases of the eye; which, according to the German method, are treated of *subjectively* and *objectively*. However copious the work may be, it would have been more complete had the author introduced the mode of using the lens; as, by its means, certain affections of the anterior and posterior chambers of the eye, can be alone observed and discriminated.

The utility of a preliminary work of this kind for the student and junior practitioner is incontestable.

"The young student while entering upon the study of ophthalmology finds himself in a state of suspense and uncertainty as to how he should proceed. Every thing seems to float in confusion before him; he feels himself incapable of recognizing the minute changes in internal structures; of distinguishing that which belongs to one texture from that which belongs to another, that which is natural from that which is the result of present or former disease. The importance of this rudimental branch of ophthalmic study is abundantly evidenced by the fact, that many practitioners, otherwise skilful and well-informed, commit gross and irremediable errors in the treatment of ophthalmic diseases, from mere inattention to the morbid phenomena."

The author, although at times tediously circumstantial, discusses fundamentally the *objective* appearances—those which are or ought to be appreciable by the practised eye of every professed oculist. He commences, in the order of enquiry, with the accessory parts, and thence passes on to those concerned in the formation of the eyeball, as follows:—

"I. External parts surrounding the globe of the eye; *a*, supraciliary region, supraciliary arch, eyebrows; *b*, palpebræ, palpebral margin, eyelashes.

"II. Lachrymal organs: *a*, lachrymal gland, lachrymal duct, tears; *b*, lachrymal puncta, and canal; *c*, lachrymal sac; *d*, nasal duct.

"III. Integral parts constituting the globe of the eye: *a*, *external parts*, conjunctiva, sclerotic, cornea; *b*, *internal parts*, anterior chamber, iris, pupil, posterior chamber, bottom of the eye.

"IV. Examination of the entire globe as respects volume, situation, consistence, mobility, direction, aspect, configuration, regard."

*Subjective ophthalmoscopy*, or that derived from the narration and interrogation of the patient, embraces

1st, the nature and degree of vision; 2d, optical illusion; 3d, pain; and the sympathies subsisting between the eye and other parts, whether effected through the medium of the nervous, the vascular, or the lymphatic system.

Dr. Marchetti's remarks on diseases are sometimes interesting.

"There is a peculiar ill-conditioned ulcer of the cornea of traumatic origin occurring in hot seasons, and characterized by having its exterior in whole or in part covered by white flakes like snow. I witnessed an instance in which the entire surface of a large ulcer presented this remarkable white appearance, as if a delicate white pellicle had been diffused over it, and which is an infallible sign of the establishment of mortification—of the *white gangrene* of the cornea."

In reference to the various degrees of sensibility and the consequent liability to inflammatory diseases of the eye, Dr. Marchetti says that if the iris be blue the organ is endowed with but little excitability, from the lower development of the sanguineous system of the choroid and iris. Hence it is less disposed to inflammatory than to catarrhal and neuralgic affections. If, on the contrary, the iris be dark, then is there a copious supply of blood, high excitability and proneness to vascular congestion, apoplexy, and amaurosis. Is not this remark more the result of theory than observation?

The particular morbid condition denominated *friability* of the iris, met with in syphilitic, arthritic, and cachectic subjects, merits notice on account of its forming a principal contra-indication to the operation for artificial pupil. It is characterized

"By our being no longer able to discover the natural vascular circles; by the iris assuming a dirty-green wash colour; by its surface presenting bluish spots, in which the texture appears wanting; by its loss of motion; and by our knowledge of the fact of its having been subjected to a severe, long-protracted, chronic inflammation."

That severe blows on the eye are not necessarily followed by serious mischief is shown from the results of two cases recorded by the author. In one the sclerotic was ruptured close to its junction with the cornea; the subsequent inflammation was very slight, and the patient suffered only weakness and confusion of sight. In the other there was procidentia of the lens at the pupil, with no other effect than a little diminution of vision.

III. The work on *Purulent Ophthalmia*, by M. Juillard of Geneva, is a thesis, and, although not the actual production of M. Sanson, must be regarded as containing an exposition of his views and practice, and as stamped with his high authority.

M. Sanson, struck with the dangers attending the different means heretofore employed against blennorrhœal or purulent ophthalmia, and the *absolute inefficacy* of the antiphlogistic plan, conceived that, by conjoining excision and cauterization, we might derive an advantageous result. The problem for solution was the following: to destroy the secreting organ wherever it exists, and to bring into relation parts whose vital properties being differently modified are therefore less disposed to adhere the one to the other.

Instead of attempting to restore the urethral discharge, which he has never seen altogether suppressed, M. Sanson practises, in the first place, a full bloodletting of from twenty-four to thirty ounces; not with the view of arresting the course of the affection, but of counteracting symptoms that might be developed, in consequence of a painful operation he proposes to perform and upon which he does not lightly decide. If contrary to expectation this primary evacuation be followed by a marked melioration of the symptoms, a fresh quantity of blood is abstracted and to a greater amount than the preceding time, leeches are at the same time applied around the orbits; in a word, the antiphlogistic method is pursued with great vigour. But, if the ophthalmia instead of abating under the influence of such treatment goes on increasing in intensity; or, remaining stationary, does not seem disposed to yield to a treatment energetic proportionately to the constitution of the sufferer, M. Sanson then renounces a procedure inappropriate to the nature of the malady, and resorts to the excision of the conjunctiva. This he performs in the following manner: the patient reclining upon a bed, one assistant keeps the head fixed upon pillows, while another separates the eyelids from each other, holding them fully everted at the same time. The surgeon then seizes with a pair of dissecting forceps the salient portions of the ocular conjunctiva, and snips away, with scissors curved on the flat as completely as possible, all the tumefied parts of that membrane, as far as and including the conjunctiva to its point of reflexion: it is, however, frequently difficult to push the operation so far. The surgeon then permits a flow of blood more or less considerable; and when he believes a sufficient local emission to have taken place he carefully wipes away the blood mingled with pus, bathing the parts, and passes a pencil of nitrate of silver over the whole internal surface of both eyelids held everted. So soon as the cauterization is at an end, the surgeon, maintaining as far apart as possible the eyelids, is to wash freely with water the cauterized membrane, in order to remove any nitrate of silver remaining uncombined with the tissues, which might adhere to the cornea and alter its transparency.

The success of the operation now described depends in a great measure on the care employed in cauterizing to a suitable degree the palpebral conjunctiva. For this purpose, we must pass lightly the pencil of lunar caustic, endeavouring to make it act more particularly on those points where the granulations are most developed and numerous. If the conjunctiva still presents folds, we must try to introduce the nitrate of silver so as to repress as far as possible all the papillary bodies there concealed. The patient should be bled, and have his feet bathed in warm water containing mustard several times a day; in fact, he is to be treated as in ordinary cases of ophthalmia. Great benefit will be obtained from the application of cold, which acts here as an astringent. The pain, very intense at the moment of the operation, is allayed so soon as the caustic touches the conjunctiva. Were we to judge of the violence of the symptoms that might be developed, from the acuteness of the pain felt during the operation, we should have much to dread; but, happily, experience has seldom justified these grave anticipations. Cauterization even to a considerable extent has been practised at the ophthalmic clinic of the Hôtel Dieu of Paris, with the nitrate of silver, without any subsequent inflammatory mischief; so true is this that M. Sanson, who at the outset never failed to prescribe, immediately after cauterizing, abundant bloodlettings both general and local, has never recourse to them now, unless there be some special indication present to necessitate their employment. M. Sanson believes that by pursuing this method not only do we fulfil the essential therapeutic indications, but we save moreover the patient the evils incident to other plans of treatment.

In reference to the indications, it may be remarked that the secreting organ is removed in certain points and cauterized in others. Secretion becomes then impossible. And if, as Jüngken affirms, the danger in blennorrhœal ophthalmia consists particularly in the acrid and corrosive qualities of the matter furnished by the papillary bodies, we shall thus have exactly fulfilled the leading indication in their obliteration.

It may be said in the second place that M. Sanson's method exposes the patient less than most others to serious consecutive symptoms. In effect, two surfaces are brought into contact whose vital properties are very different. One of these, the bleeding surface, will cicatrize more rapidly than the other, presenting an eschar profound in proportion to the action of the caustic; there will be developed in this last an *eliminatory* inflammation which, requiring a longer or shorter time to run its course and detach the eschar, will expose a denuded surface at the period only when the process of cicatrization is complete in the other. It may be supposed that the coalition of the eyelids with the eyeball will be much less likely to occur, if it be possible, under the operative procedure.

Lastly, this mode of treatment is in certain instances the only one applicable. When, for example, after a patient has been submitted to a rigorous treatment, the other eye becomes in its turn affected, it is evident that copious depletions proportioned to the gravity of symptoms can no longer be resorted to, without compromising the patient's existence. It was this circumstance which suggested to M. Sanson, at the bedside of a patient, the idea of excising all that was possible of the conjunctiva, and destroying the secreting organs beyond the reach of surgical manipulation. This plan of treatment, or some other analogous, will be alone admissible when we have to deal with subjects advanced in years or of a feeble constitution. It is well known that sanguine emissions, even to a limited extent, may under such circumstances prove highly prejudicial.

Two cases of gonorrhœal ophthalmia are reported by M. Juillard, in which the above method effected a speedy cure.

IV. We have introduced into our list of books at the head of the present article, the first Part of the Cyclopædia of Surgery, for the purpose of noticing the Treatise on Amaurosis contained in it. This, the production of Mr. Tyrrell, is by far the best article in the fasciculus, and is highly creditable to its excellent author. We do not agree with Mr. Tyrrell in all respects, and we shall not hesitate to state the points whereon we differ from him: but we strongly recommend his paper to the attention of all ophthalmological surgeons, as a work of great practical value.

Mr. Tyrrell sets out with a remark, which might lead the reader, were he not otherwise better informed, to suppose that the term Amaurosis had at one period comprehended all the diseases which obscure the sight; whereas it is a word introduced by the later Greek writers as synonymous with the amblyopia of Hippocrates and the melania of Aristotle, names which were always limited to affections of vision independent of visible change behind the pupil. A good historical dictionary of medical terms is a desideratum: well executed, it would afford much amusement as well as instruction, and prevent unlearned writers from falling into many sad blunders.

After explaining that amaurosis may result from disease in the retina, the optic nerve, or the brain, Mr. T. observes that "from the known principles of nervous power, it must be obvious that any disease affecting the cerebral portion of this nervous apparatus, or the optic nerve, must influence the extreme part of the nerve, or the retina, so that disease cannot exist in either of the former without producing a diminution or destruction of the functions of the latter." (P. 77.) This statement, if left unqualified, might mislead. That disease in the brain, or in the optic nerve, may obliterate the power of vision, is readily granted; but that disease in either, even to the extent of complete blindness, necessarily affects the peculiar functions of the retina, we apprehend to be an error. The functions of the retina are to receive the impressions of light and transmit them to the optic nerve; and these functions may be perfectly performed, although, from disease in the brain or in the optic nerve, no perception of light is retained. Nay, farther, the functions of the optic nerve may be perfectly performed, along with those of the retina, although, in consequence of disease existing in the tubercula quadrigemina, the patient may be totally amaurotic. The function of the optic nerve is to transmit the impressions made on the retina to two quarters: first, to the third pair of nerves, by which the iris is moved; and secondly, to the brain, where alone visual perception is effected: and we sometimes find the former part of the function of the optic nerve performed and the pupil moved truly according to the degrees of light to which the eyes were exposed, although the latter part is null and the patient totally blind.

Mr. Tyrrell adopts the old distinction of amaurosis into functional and organic; the former division comprehending, as he says, "those which are not attended with any appreciable change in structure," and the latter "those which are attended with some change." (P. 77.) It is plain that every thing regarding this distinction hinges on the meaning given to the words "appreciable" and "structure." Now, ninety-nine out of one hundred inspectors of dead bodies, on looking at a retina affected with neuromata, would in all likelihood discover nothing wrong in it. It would only be the cautious microscopic observer who would detect these minute tumours, scattered through the medullary layer of that membrane. Thousands of pathological changes not yet appreciated, are no doubt appreciable, and will yet be appreciated.

Then, as to "structure:" are changes in the blood-vessels and in the blood to be deemed structural changes or not? Is the term "organic disease" to be restricted to those morbid alterations in which there is an obvious and *permanent* change in structure; or are we to extend the meaning of the phrase, and include the primary and secondary changes resulting from inflammation? Not long ago we drew blood from a patient labouring under arthritic amaurosis; and, on submitting a little of that blood to the microscope, it was evident that the red globules were deficient in quantity and unnatural in their appearance. This surely was an organic change, although without this peculiar mode of examination no such change could have been appreciated. What Haller remarked regarding disorders of the mind, is still more applicable to the subject before us. "Id utique adparet, plerumque in mentis vitiis encephalum pati: et si aliquando rariori exemplo non visum est pati, *potuit vitium in minoribus elementis latuisse, aut incisori patientia defuisse.*" (*Elem. Phys.*, v. 574.)

At page 95, Mr. Tyrrell even talks of functional disturbance of the orbital portion of the optic nerve from the effect of pressure, perhaps absorption, of the nerve, from exostoses and other tumours. Surely Mr. T. cannot suppose that the contact of a morbid growth in the cavity of the orbit will destroy the function, without affecting the material elements of the optic nerve. If any disorder to which the human frame is subject deserve the name of an organic disease, it is such a one as this, where a tumour or an exostosis, perhaps palpable to the observer even during the patient's life, and perfectly appreciable after death, prevents the nutrition of the optic nerve, compresses its medullary substance, and at last causes its absorption.

Besides the distinction of functional and organic, the following classification of the particular varieties of amaurosis has been adopted by Mr. Tyrrell:—I. Functional amaurosis of retina. II. Organic amaurosis of retina: 1, from inflammation of the retina, acute and chronic; 2, from inflammation of the choroid, acute and chronic; 3, from inflammation of the globe generally; 4, from lesions of the globe; 5, from scrofulous and malignant disease of the retina. III. Functional amaurosis of optic nerve. IV. Organic amaurosis of optic nerve. V. Functional amaurosis of cerebrum. VI. Organic amaurosis of brain or its membranes. VII. Functional amaurosis from excess of vascular action. VIII. Functional amaurosis from deficient supply of red blood. IX. Congenital amaurosis.

Under these heads, Mr. Tyrrell has communicated much useful information on the symptoms, causes, and treatment of the various affections of which he speaks. The treatment which he recommends is on the whole highly judicious, and his observations on diagnosis are particularly valuable. "I have alluded," he says, "to the close resemblance which exists between some of these cases, [cases of asthenic amaurosis,] and those in which the amaurosis is caused by cerebral pressure, and the consequent liability to mistake in diagnosis and treatment." This point he illustrates by cases, one of which we cannot forbear quoting.

"A young and delicate female, about seventeen years of age, having great nervous susceptibility, and disposed to hysteria, was accidentally thrown from a gig with her father, who was a very large man. She fell upon her father, and was taken up without any mark of injury: she was however dreadfully alarmed, and soon after attacked with a violent fit of hysteria: several hours elapsed before she could be roused from this under the use of the ordinary treatment, by stimuli, anti-spasmodics, &c. She then complained of excessive headach, with giddiness, confused vision, and heat of head; the stomach was irritable; she suffered from great mental depression, and could not procure sleep. Whilst in this state her medical attendant (believing that she had received some concussion of brain by her fall) bled her: she fainted after the loss of about eight ounces of blood, and remained a long time in a condition approaching syncope. In a few hours afterwards her previous symptoms were renewed and became much aggravated, the headach almost intolerable, the depression excessive, and the affection of vision increased, so that in forty-eight hours after the venesection she could not discern light. A few hours after the amaurosis was complete, she was brought to me, still labouring under the symptoms last mentioned; but, in addition, I found her pallid, with cold extremities, and a quick, feeble, and easily compressible pulse: the result of the abstraction of blood was sufficiently indicative of the nature of the affection, and to prove its asthenic character. I directed perfect quiet, the recumbent posture, small portions of nutritious matter to be given frequently, ten drops of the vinum ferri to be taken in weak wine and water every four hours, and an occasional dose of the compound decoction of aloes with manna, and a few drops of aromatic spirit of ammonia as an aperient. In forty-eight hours from the time I prescribed for her, her symptoms were greatly mitigated, and she began to perceive light again; she soon recovered her ordinary condition of health, and lost her headach, feeling of depression, &c., but the functions of the retina returned very gradually; so that several weeks elapsed before she could tell the letters in the title-page of a common octavo work; being so far restored, I lost all knowledge of her for several months, when she was again brought to me nearly in the same state as when I first saw her: her father had failed in business, and she had suffered much from anxiety and privation, and had neglected all treatment. I put her under the same plan of treatment again, gradually increasing the dose of steel, and occasionally varying the form of medicine: her health was rapidly re-established, and a very slow improvement took place in vision; but after many months' careful perseverance in the above means she obtained only sufficient vision to be able to make out a large print. I subsequently tried blistering, electricity, galvanism, and strychnine, but without any further benefit." (P. 102.)

With regard to the treatment of the different varieties of amaurosis, Mr. T. very properly regulates it according to the particular symptoms present in each case, and gives little encouragement to such general remedies as emetics, arnica, phosphorous, strychnine, and the like. "I have frequently tried the influence," says he, "of strychnine in cases of amaurosis, which I considered most appropriate for its use; but I have been very greatly disappointed in its effects: I have not seen one single instance of benefit from its employment in the manner generally recommended." (P. 105.)

The first variety of amaurosis, according to Mr. Tyrrell's classification, is functional amaurosis of the retina; which he believes may arise from one or other of these four causes, viz. non-exercise of sight, excessive light, concussion of the eye, and narcotic poisons. Mr. Tyrrell thinks that a squinting eye becomes amaurotic because it is not in use; the common notion is probably the more correct, that the eye being defective is not used. That, by looking steadily at the sun, a person may lose his sight, or that the same result may follow a blow on the eye, will not be denied; but that such effect will arise without inflammation, or some other organic lesion of the retina, is a thing not to be credited. Belladonna applied to an ulcer on any part of the body, or taken internally in a sufficient dose, will enormously dilate the pupil, and thereby render vision for a time indistinct. But this is not amaurosis. This is merely mydriasis; and by making the patient view objects through a small hole, we have always found, in such cases, that the retina completely retained its sensibility.

Under the title of "Retinitis, acute and chronic," Mr. T. has introduced a description of the disease well known to oculists as acute and chronic glaucoma; and under that of "Choroiditis," he has described amaurosis resulting from internal scrofulous ophthalmia, or what Rosas calls scrofulous amaurosis. This changing of names is quite intolerable; and we are sorry that Mr. Tyrrell has added to the evil. In fact, it would require years of study to become acquainted with the mere synonymes, which have been heaped up to such a Babelish height by the writers on eye-diseases. What Mr. Lawrence terms acute glaucoma, Mr. Tyrrell calls acute retinitis. Again, Dr. Mackenzie has described a very peculiar disease of the eye, under the name of choroiditis. His description has been borrowed by Mr. Middlemore, and adopted (with due acknowledgment) by Dr. Sichel. We believe Dr. Mackenzie to be quite wrong in calling this disease choroiditis; it is neither more nor less than the sclerotomalakia of the Germans. But here we have Mr. Tyrrell with an entirely new application of the same term. His choroiditis is nothing more than scrofulous iritis, or internal scrofulous ophthalmia, ending in blindness. Moreover, we are not at all satisfied that glaucoma is essentially an inflammation of the retina; nor that internal scrofulous ophthalmia is so much an affection of the choroid as it is of the retina and iris. Dissections only could warrant Mr. Tyrrell's views of glaucoma and scrofulous amaurosis; but he makes no appeal to evidence of this sort. Certain we are that chronic glaucoma is not a retinitis merely. In fact, in this disease, the retina is often the last part to be affected.

Mr. Tyrrell commences his description of internal scrofulous ophthalmia or chronic choroiditis, by saying that more or less functional disturbance usually precedes the inflammatory action in the choroid. The disturbance he here refers to, consists in the symptom commonly called *muscæ volitantes,* a symptom which in all probability depends almost constantly on a dilated state of the retinal vessels; pressure on the convex surface of the retina gives rise to photopsia and prismatic vision, not to muscæ volitantes. This is, at least, the general opinion; and it remains with Mr. T., when opportunity offers, to explain the grounds of an assertion so contrary to the common belief as that this symptom depends on the state of the choroid.

Mr. T. speaks of volitant and fixed muscæ, but neglects to draw any accurate distinction between them, or to explain the cause of the difference, if difference there be. It is well known, that, from the cause of muscæ rarely existing in the axis of vision, they appear to fly upwards or downwards, or from one side to the other, whence the appellation volitantes. These we presume are the volitant muscæ of Mr. T. What, then, are the fixed—and what are the evanescent,—which seem to be a third variety, also left without any accurate distinction? He adopts the popular view of the exciting cause of muscæ, that they depend on the stomach. He might as well say the acidity and other stomachic ailments depended on the muscæ. Cerebral disease is very frequently the cause of dyspepsia; and along with this the patient is affected with muscæ, and perhaps slight amblyopia. A congestive state of the brain, producing the dyspepsia, is attended by a similar condition of the retina, which causes the muscæ.

We observe that in speaking of cataract and its diagnosis from glaucoma (p. 83,) Mr. Tyrrell states that the cataractous opacity occupies, in ordinary cases, the central part of the lens. If he means the centre of the lamella forming the anterior surface of the lens, we shall not dispute the matter; but if he means the kernel or centre of the lamellæ generally, we are positive cataract never commences there, but always superficially. We have examined many cataractous lenses immediately after extraction, and in ninety out of the hundred only a few of the exterior lamellæ were affected with the peculiar opaque condition, called cataract; the rest being generally amber-coloured and pretty transparent. In this respect, the cataractous degeneration of the lens is the reverse of the glaucomatous; for the latter affects chiefly the lamellæ immediately behind the kernel of the lens.

Although we enter our protest against the change of names which Mr. Tyrrell has introduced, and must object to the introduction of his *retinitis* and *choroiditis* under the head of amaurosis, still we most willingly testify to the truth and clearness of his descriptions, as well as to the prudence and skilfulness of the treatment which he advises. In both diseases, he trusts much to mercury; and the plan of establishing a full and free mercurial action, and sustaining it for several weeks or months, which was so beneficial in the hands of Heister, appears in those of Mr. T. to have also proved successful even in cases of complete amaurosis.

"One of the first cases of this kind that came under my care was in a powerful man about thirty-eight years of age, from the north of Ireland, who was sent to me by my late excellent friend, Dr. Babington: he had been amaurotic for seven years, and had lost the perception of light; but the globes possessed their natural firmness and elasticity; the pupils were clear but irregular, from many points of adhesion between the pupillary margin of the iris, and the anterior capsule of the lens; the irides were discoloured and dull, and he had the vacant aspect of a blind person. I admitted him into the infirmary, (then in Charterhouse square,) and put him under mercurial treatment, with a nutritious diet: as soon as the mouth became tender, a considerable degree of sclerotitis occurred, with pain and tenderness of the eyeballs; the plan was, however, steadily continued, and some belladonna was applied night and morning to each eyebrow; he soon became sensible of light, and gradually acquired the power of discerning surrounding objects, and at the same time the adhesions between the iris and the capsule of the lens began to give way, and the pupils to reassume their natural figures; by degrees the vision improved, all appearance of inflammatory action subsided, the pupils became nearly regular, and the irides brilliant; the full mercurial action was kept up for above sixteen weeks, when the amaurosis was completely subdued, and his vision perfect. For sixteen weeks he discharged about a pint and a half of saliva daily; but, in spite of the severe treatment, he came out of the course improved in appearance, and evidently increased in bulk." (P. 92.)

Organic amaurosis from disease of the brain or its membranes is of course a wide field; presenting great variety in the symptoms, according to the seat, nature, and extent of the morbid changes. In some cases, it assumes the form of hemiopia, in others the whole field of vision is obscured. In some the pupil is dilated and fixed, in others it moves freely. Of this last remarkable fact, Mr. T. attempts no explanation. Double vision, strabismus, and palsy of the muscles of the eyeball and eyelids, are symptoms noticed particularly by Mr. T. as occasionally attendant on this variety. The chief immediate causes which he indicates, are injuries to the head, fevers, intemperance, and excessive mental labour. The treatment is comprised under these three heads: relief of vascular turgescence by general and local bleeding; prevention of further congestion or turgescence by diet, mental and bodily quiet, and counter-irritation; promotion of absorption of morbid deposits, by counter-irritants, mercury, iodine, &c.

Mr. T.'s account of congestive amaurosis, and of that arising from deficiency of red blood, is plain and sensible. He winds up the subject with some hints regarding the pathological anatomy of the disease well deserving of attention. Many of the changes in structure which we find in our examinations of amaurotic cases, he believes to be the effect, and not the cause, of the loss of vision. This happens especially with respect to the optic nerve, which almost always loses its natural character under continued amaurosis, whatever has been its origin.

Mr. Tyrrell concludes the Article with some good remarks on feigned amaurosis, which he has sometimes succeeded immediately in detecting by the following means:

"During conversation dropping some small object, as a knife or pencil suddenly, which has been immediately picked up by the patient—pretending to see something in the room, or out of the window (if near) of curious or unusual character, the patient has been unguarded for a moment, and his eyes have followed the direction I have pointed to; asking how the patient's dress became torn or dirtied, his eyes have immediately been directed to the part mentioned or pointed to; and several other like expedients. In one case, of a little girl, which baffled me for two or three weeks, during which period she had been strictly watched, but nothing elicited; I was engaged in conversation with her about her medicines, which she had much abhorrence of, and after trying to persuade her to take them well, I said I would give her sixpence if she would promise to do so, she assented, and I held out a halfpenny towards her, which she directly said (without touching it) was not a sixpence: she had previously sat for hours together without moving, and would allow me to place my finger or other matter in contact with the cornea without flinching." (P. 107.)

V. Our limits will not allow us to notice the small work of Dr. Littell in detail; but, after an attentive perusal of the whole volume, we confidently recommend it to the senior as well as junior members of the profession. It is replete with information, yet so terse in style and compressed in bulk as at once to entice and repay perusal. We agree in most points with the author's pathological inductions and practical precepts. The language is free from any tinge of Americanism, the descriptions are short but comprehensive, while the treatment is characterized by great prudence; on the one hand, avoiding the charge of inactivity or feebleness, on the other, never risking the more serious results of chronic mischief and broken health from excessive depletion, or the depressing effects of violent mercurial courses; of which faults some of our own countrymen are not entirely innocent.

It is no small triumph to Dr. Littell to be able to say that he has introduced almost all that is valuable, and every thing absolutely necessary to the student, within the compass of 250 small pages; and we would deliberately recommend our young friends to read this work before encountering the voluminous treatises of Lawrence, Travers, Mackenzie, Middlemore, &c. We are in no way inclined to speak slightingly of these works: on the contrary, we believe that there is much accurate observation, learned research, and sound practice to be found in them: but the commencing enquirer is startled by their magnitude, and discouraged by the belief that the subject of ophthalmology ought indeed to be cultivated by exclusive practitioners, when he finds such extensive and elaborate treatises devoted to its consideration. Apprehensions and misapprehensions of this sort the small volume before us is well calculated to remove, and we once more earnestly recommend it to the attention of the student.

---

